# Supplementary material for: Machine Learning-Assisted Classification of Paraffin-Embedded Brain Tumors with Raman Spectroscopy
Source: Brain Sci. 2024 Mar 23;14(4):301. doi: 10.3390/brainsci14040301 (PMC11048578; doi:10.3390/brainsci14040301)
Supplement: Supplementary file 1 [file brainsci-14-00301-s001.zip › brainsci-2892164-supplementary.pdf]

Supplementary Material

Preprocessing

Cosmic ray artefacts were removed from individual recordings with a median filter across replicated acquisitions. A trend removal was performed using an iterative use of a custom piecewise Savitzky-Golay filtering and morphological opening of the signal to avoid removing crucial information from the spectrum. Spectra were then cropped in the morphological region (between $500cm^{-1}$ and $2000cm^{-1}$ on the Raman shift axis) and standardized individually (mean removal and divided by the standard deviation).

Machine Learning Pipeline: Data preparation

The dataset has been split into a training set and a test following a tailored selection taking into account the distribution of patient count in each of the tumors specimen. The aim of this specific split is to assess the generalization of the model over each subcategory of tumors (astrocytomas, oligodendrogliomas, ependymomas, glioblastomas, transitional/meningothelial meningiomas, breast carcinoma, colorectal carcinoma, non-small cell lung carcinoma (NSCLC)), even to model the discrimination of the main category (meningioma, carcinoma, glioma). Hence, the split was performed patient-wise for each subcategory with approximately 75% of the patient count remaining in the training set.

Machine Learning Pipeline: Choice of model

The performance of several machine learning algorithms was compared with internal cross validation (randomly stratified internally, 4 splits, repeated 3 times), together with a set of hyperparameters for each of them (optimized on a grid). The methodology considered linear and nonlinear algorithms, those were: Logistic Regression, Random Forest, Support Vector Machine, K-Nearest Neighbors. The hyperparameters grid considered respectively:

Logistic Regression:

- Strength of the regularization parameter for L1/L2/ElasticNet [0.5,0.75,1]
- Weighted/Unweighted cost function

Random Forest:

- Max depth of the trees [2,3,10,None]
- Minimum sample to consider a leaf [1,2]
- Minimum samples to consider a split [5]
- Number of trees [50,75,100,125,150]
- Weighted/Unweighted cost function

Support Vector Machine:

- Linear Kernel
- C parameter [0.5,1,5,10]
- Weighted/Unweighted cost function

K-Nearest Neighbors:

- Number of neighbors [2,5,10,20]
- Weights [Uniform, Distance]
- Weighted/unweighted cost function
- Leaf size [15,30,45]
- P parameter [1,2]

The aforementioned modeling pipeline has been applied on each classification task and Random Forest estimators consistently outperformed its competitors, albeit with varying hyperparameter sets.

Machine Learning Pipeline: Feature Importance

Feature importance was estimated from the mean decrease in impurity within each tree.

Supplementary Material – Figures

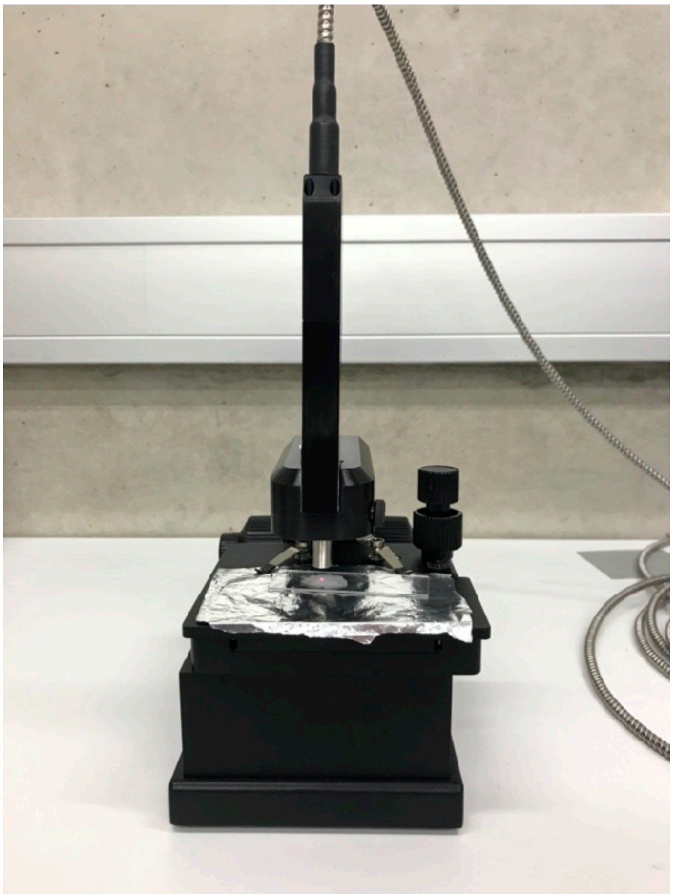

Supplementary Figure S1 Raman Spectrometer set up during measurement. For sake of easier depiction, the dark chamber is not shown here.

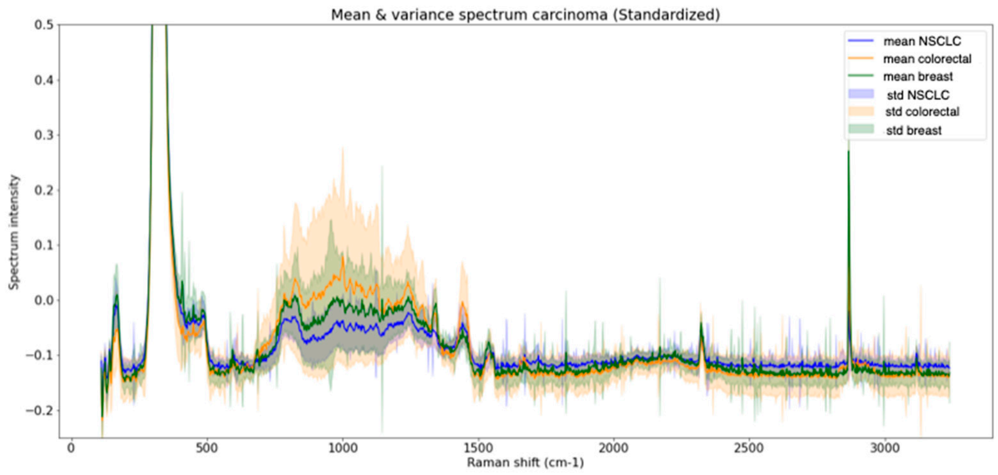

Mean & variance spectrum carcinoma (Standardized)
mean NSCLC
mean colorectal
mean breast
std NSCLC
std colorectal
std breast
Spectrum intensity
Raman shift (cm-1)
0.5
0.4
0.3
0.2
0.1
0.0
−0.1
−0.2
0
500
1000
1500
2000
2500
3000

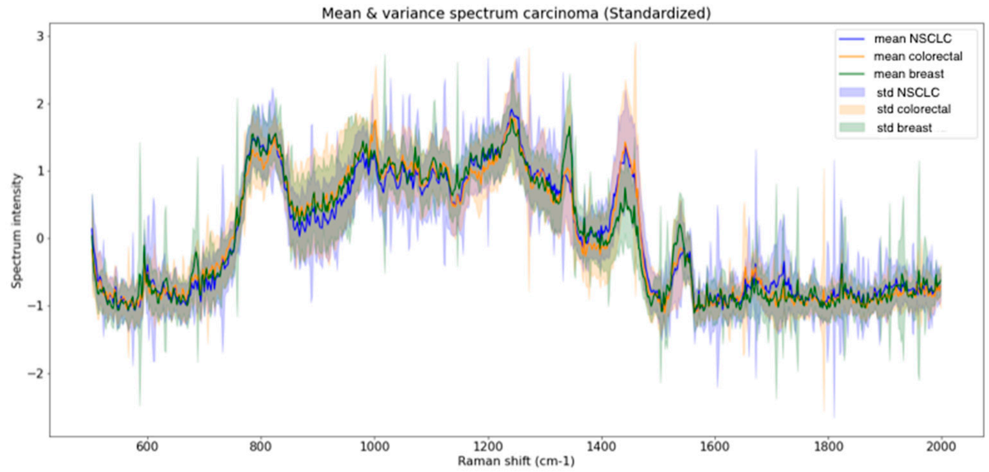

Mean & variance spectrum carcinoma (Standardized)
mean NSCLC
mean colorectal
mean breast
std NSCLC
std colorectal
std breast
Spectrum intensity
Raman shift (cm-1)
3
2
1
0
−1
−2
600
800
1000
1200
1400
1600
1800
2000

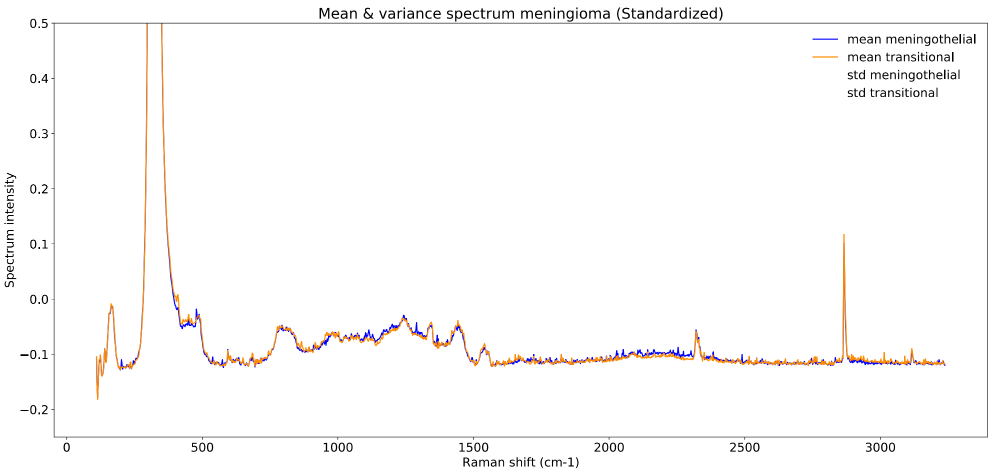
Mean & variance spectrum meningioma (Standardized)
mean meningothelial
mean transitional
std meningothelial
std transitional
Spectrum intensity
Raman shift (cm-1)

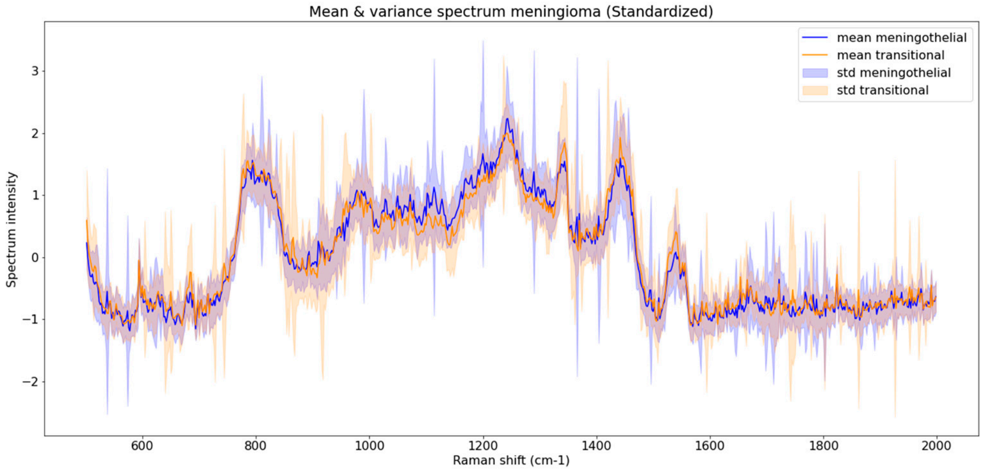
Mean & variance spectrum meningioma (Standardized)
mean meningothelial
mean transitional
std meningothelial
std transitional
Spectrum intensity
Raman shift (cm-1)

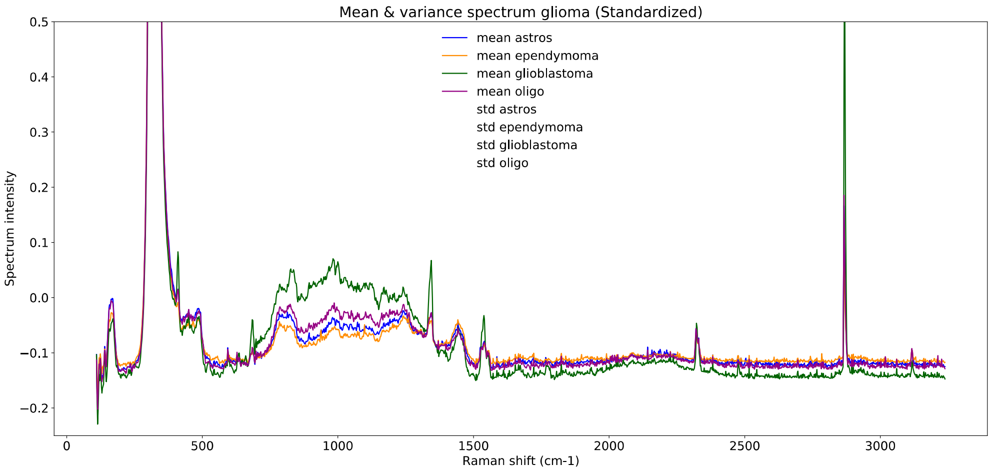
Mean & variance spectrum glioma (Standardized)
mean astros
mean ependymoma
mean glioblastoma
mean oligo
std astros
std ependymoma
std glioblastoma
std oligo
Spectrum intensity
Raman shift (cm-1)

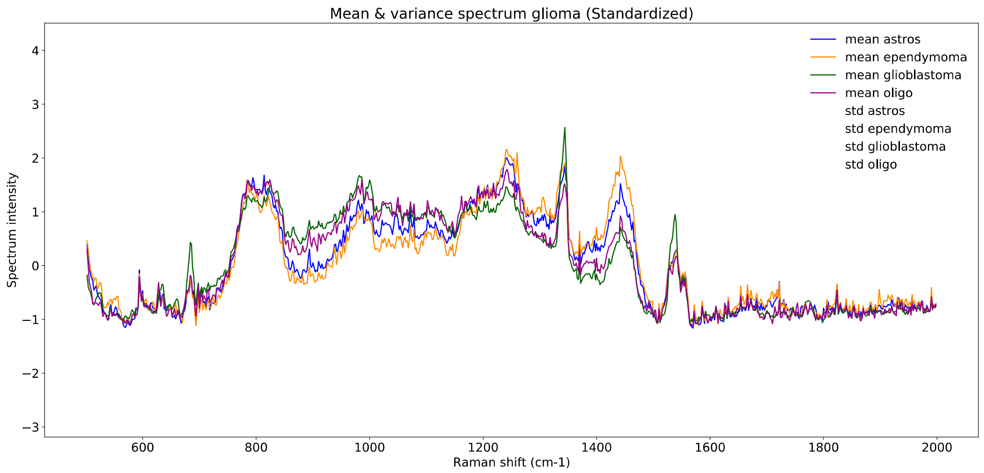

Supplementary Figure S2 displays all raw Raman Spectra plus Raman spectra cropped within the “biological” wavenumber region 500cm^{-1} and 2000cm^{-1} below - sorted by tumor entity.

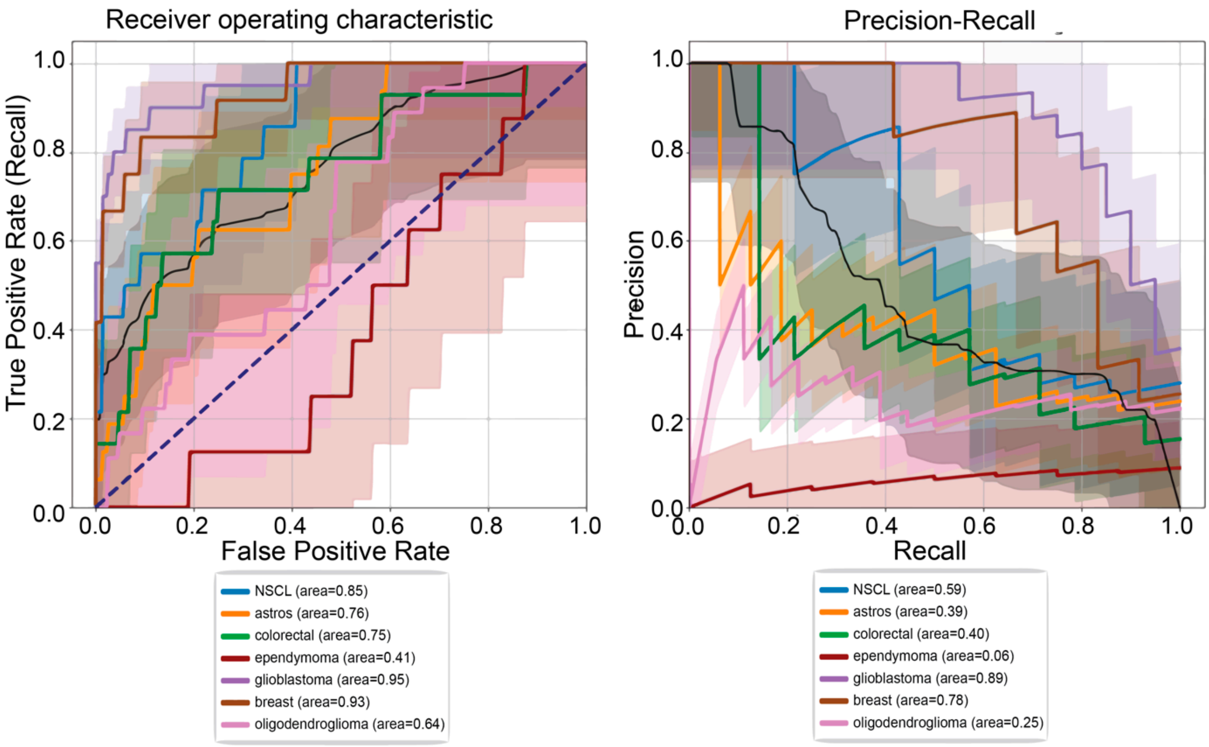

Supplementary Figure S3. ROC and PR curves as well as AUROC and AUPR values of the 7-class learning approach.

Accuracy: 0.32
Detail:

	precision	recall	f1-score
NSCLC	0.36	0.57	0.44
astrocytoma	0.23	0.69	0.35
colorectal	0.00	0.00	0.00
ependymoma	0.00	0.00	0.00
glioblastoma	0.75	0.30	0.43
breast	0.75	0.50	0.60
oligod.glio.	0.25	0.11	0.15
accuracy			0.32
macro avg	0.34	0.31	0.28

Supplementary Figure S4. The metrices precision, recall, and f1-score of the 7-class learning approach are displayed.

Accuracy: 0.68
Detail:

	precision	recall	f1-score
NSCLC	0.54	0.50	0.52
colorectal	0.59	0.71	0.65
mamma	1.00	0.83	0.91
accuracy			0.68
macro avg	0.71	0.68	0.69

Supplementary Figure S5. The metrices accuracy, precision, recall, and f1-score of the carcinoma brain metastases classifier are displayed.

Accuracy: 0.53
Detail:

	precision	recall	f1-score
astros	0.57	0.72	0.63
ependymoma	0.41	1.00	0.58
glioblastoma	0.80	0.19	0.31
oligo	0.58	0.50	0.54
accuracy			0.53
macro avg	0.59	0.60	0.52

Supplementary Figure S6. The metrices accuracy, precision, recall, and f1-score of glioma classifier are displayed.

Accuracy: 0.92
Detail:

	precision	recall	f1-score
carcinoma	0.82	0.93	0.88
glioma	0.97	0.92	0.94
accuracy			0.92
macro avg	0.90	0.93	0.91

Supplementary Figure S7. The metrices accuracy, precision, recall, and f1-score of binary necrosis classifier are displayed.

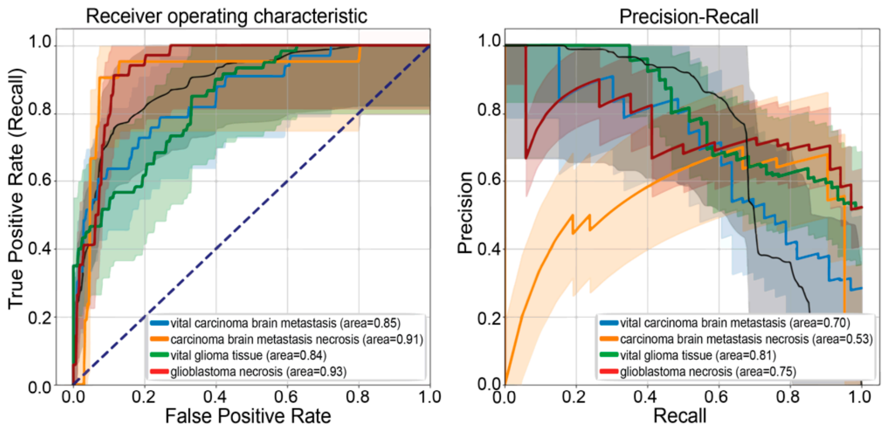

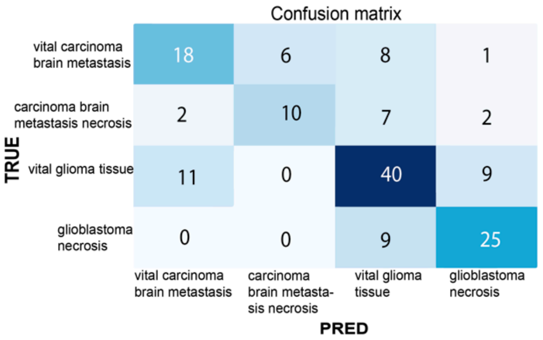

Accuracy: 0.63
Detail:

	precision	recall	f1-score
vital carcinoma met.	0.58	0.55	0.56
carcinoma met. necrosis	0.62	0.48	0.54
vital glioma tissue	0.62	0.67	0.65
glioblastoma necrosis	0.68	0.74	0.70
accuracy			0.63
macro avg	0.63	0.61	0.61

Supplementary Figure S8. ROC and PR curve, confusion matrix, as well as the metrices accuracy, precision, recall and f1-score of multiclass vital area vs. necrosis classifier are displayed.

	FrequencyBinsFeatures	Importance
0	682.0	0.008510
1	918.0	0.007418
2	1412.0	0.007313
3	1030.0	0.007168
4	1524.0	0.006880
5	808.0	0.006739
6	1526.0	0.006531
7	1434.0	0.006487
8	1044.0	0.005974
9	1450.0	0.005758

	FrequencyBinsFeatures	Importance
0	684.0	0.016167
1	682.0	0.014295
2	1530.0	0.014041
3	686.0	0.013992
4	1536.0	0.013743
..	...	...
95	1082.0	0.001832
96	1294.0	0.001828
97	1254.0	0.001825
98	520.0	0.001811
99	996.0	0.001806

Supplementary Figure S9. Listed Raman bands and corresponding feature importance (FI) for classification is shown using two selected examples. Left: FI for the glioma classifier. Right: FI for the multiclass vital area vs. necrosis classifier.

Supplementary Material – TABLES

Supplementary Table S1. Clinical information of measured glioma samples.

sample number	tumor entity	age	sex	localization	features
1	pilocytic astrocytoma, grade 1	11	male	posterior fossa	flat copy number variation profile, no mutation in analyzed regions from BRAF, IDH1, IDH2
2	diffuse astrocytoma, grade 2	22	male	left Wernicke area	IDH mutant, MGMT methylated
3	diffuse astrocytoma, grade 2	25	male	left frontal	IDH mutant, MGMT methylated
4	diffuse astrocytoma, grade 2	41	female	right temporal	IDH mutant, gain of chromosome 7, partial loss of chromosome 13
5	diffuse astrocytoma, grade 2	44	male	right temporal	IDH mutant, MGMT methylated
6	anaplastic astrocytoma, grade 3	33	female	left temporal	IDH mutant, MGMT methylated, partial losses of chromosome 3,5,9,11,13 and 22
7	anaplastic astrocytoma, grade 3	32	male	cerebral	IDH mutant, no CDKN2A/B deletion found

8	anaplastic astrocytoma, grade 3	61	male	temporo-frontal	IDH mutant, MGMT methylated, loss of chromosome 15, partial loss of chromosome 3 and 4, gain of chromosome 7 and 8
9	anaplastic astrocytoma, grade 3	35	female	left parietal	IDH mutant, MGMT methylated, partial gains on chromosomes 7q and 12p.
10	oligodendroglioma, grade 2	56	male	fronto-temporo-insular	IDH mutant, MGMT methylated, 1p/19q codeletion
11	oligodendroglioma, grade 2	24	male	left insular-opercular	IDH mutant, MGMT methylated, 1p/19q codeletion
12	oligodendroglioma, grade 2	38	male	left temporal	IDH mutant, MGMT methylated, 1p/19q codeletion
13	oligodendroglioma, grade 2	57	female	left insular	IDH mutant, MGMT methylated, 1p/19q codeletion
14	anaplastic oligodendroglioma, grade 3	44	female	left frontal	IDH mutant, MGMT methylated, 1p/19q codeletion
15	IDH mutant high-grade glioma grade 3	35	female	left temporal	IDH mutant, MGMT unmethylated, loss of 10, 11 and 19q, gain of chromosome 8

16	anaplastic oligodendroglioma, grade 3	33	male	right temporal	IDH mutant, MGMT methylated, 1p/19q codeletion
17	myxopapillary ependymoma	50	female	L1-L3	not specified
18	ependymoma, grade 2 (spinal)	50	female	intramedullar	MGMT unmethylated, partial losses of chromosome 1, 13, 14, 21 and 22
19	ependymoma with focal features of a subependymoma, grade 2	75	female	right intraventricular	MGMT methylated, partial loss of chromosome 13q, gain of chromosome 5
20	anaplastic ependymoma, grade 3 (spinal)	38	male	intramedullar Th1-Th5	MGMT unmethylated, multiple chromosomal gains and losses
21	anaplastic ependymoma, grade 3 (supratentorial)	21	male	right frontal	MGMT unmethylated, RELA fusion positive
22	glioblastoma, grade 4	50	female	bifrontal	IDH wildtype; subclass RTK II; MGMT methylated
23	glioblastoma, grade 4	66	male	left frontal	IDH wildtype; subclass RTK II; MGMT methylated
24	glioblastoma, grade 4	62	male	left temporal	IDH wildtype; subclass RTK II; MGMT unmethylated
25	glioblastoma, grade 4	61	male	right fronto-temporal	IDH wildtype; subclass mesenchymal; MGMT methylated
26	glioblastoma, grade 4	57	male	right frontal	IDH wildtype; subclass RTK I; MGMT unmethylated

27	glioblastoma, grade 4	56	male	left occipital	IDH wildtype; subclass mesenchymal; MGMT not interpretable
28	glioblastoma, grade 4	62	male	right frontal	IDH wildtype; subclass mesenchymal; MGMT unmethylated
29	glioblastoma, grade 4	72	female	right temporal	IDH wildtype; subclass RTK II; MGMT methylated
30	glioblastoma, grade 4	60	male	left temporal	IDH wildtype; subclass RTK II; MGMT unmethylated
31	glioblastoma, grade 4	54	male	right frontal	highest score: IDH, wildtype; subclass mesenchymal; MGMT unmethylated
32	glioblastoma, grade 4	74	female	right postcentral	IDH wildtype; subclass RTK II; MGMT unmethylated
33	glioblastoma, grade 4	44	female	left temporal	highest score: IDH, wildtype; subclass mesenchymal; MGMT methylated
34	glioblastoma, grade 4	50	male	right temporal	highest score: IDH, wildtype; subclass mesenchymal; MGMT unmethylated
35	glioblastoma, grade 4	70	male	left temporo-dorsal	IDH wildtype; subclass RTK II; MGMT unmethylated
36	glioblastoma, grade 4	61	male	parieto occipital	IDH wildtype; subclass RTK II; MGMT methylated
37	glioblastoma, grade 4	61	male	right fronto-temporal	IDH wildtype; subclass mesenchymal; MGMT methylated
38	glioblastoma, grade 4	54	female	left occipital	IDH wildtype; subclass RTK I; MGMT unmethylated
39	glioblastoma, grade 4	67	male	left temporo-insular	IDH wildtype; subclass mesenchymal; MGMT not interpretable

40	glioblastoma, grade 4	72	male	right opercular	IDH wildtype; subclass RTK II; MGMT methylated
41	glioblastoma, grade 4	64	male	right fronto-dorsal	IDH wildtype; subclass mesenchymal; MGMT methylated
42	glioblastoma, grade 4	69	male	left fronto-parietal	IDH wildtype; subclass RTK I; MGMT unmethylated
43	glioblastoma, grade 4	50	female	left temporal	highest score: IDH wildtype; subclass mesenchymal, MGMT methylated
44	glioblastoma, grade 4	48	male	right parietal	IDH wildtype; subclass RTK II; MGMT unmethylated
45	glioblastoma, grade 4	64	male	left temporal	IDH wildtype; subclass RTK II; MGMT methylated
46	glioblastoma, grade 4	64	female	right occipital	IDH wildtype; subclass mesenchymal; MGMT methylated
47	glioblastoma, grade 4	59	female	right parieto-occipital	highest score: IDH wildtype; MGMT non conclusive
48	glioblastoma, grade 4	54	female	left occipital	IDH wildtype; subclass RTK I; MGMT unmethylated

Supplementary Table S2. Deparaffinization protocol to reduce influence of residual wax on biological spectroscopic signal.

Deparaffinization protocol (CaF2 slide; tissue thickness 7µm)	duration
heating at 60°	60 min
Xylene bath	15 min
Xylene bath	15 min
ethanol (99%) bath	2 min
ethanol (96%) bath	2 min
ethanol (70%) bath	2 min
water bath (rehydration)	few seconds

Supplementary Table S3. Data details for training and the external validation data set split for initial 3 class analysis. Above: Training data set. Bottom: Test data set.

class	patients (n)	measurements (n)
carcinoma metastases (NSLC, colorectal, breast)	17	114
primary brain tumors (astrocytoma, ependymoma, oligodendroglioma, glioblastoma)	24	182
meningeal tumors (meningioma transitional / meningothelial)	8	72

class	patients (n)	measurements (n)
carcinoma metastases (NSCLC, colorectal, breast)	6	40
primary brain tumors (astrocytoma, ependymoma, oligodendroglioma, glioblastoma)	8	62
meningeal tumors (meningioma transitional / meningothelial)	2	20

Total number of patients, n = 65. Total number of measurements n = 490.

Supplementary Table S4. Carcinoma classifier data. Above: Training data set. Bottom: Test data set.

class	patients (n)	measurements (n)
NSCLC	7	58
colorectal	4	23
breast	6	33

class	patients (n)	measurements (n)
NSCLC	2	14
colorectal	2	14
breast	2	12

Supplementary Table S5. Glioma classifier data. Above: Training data set. Bottom: Test data set.

class	patients (n)	measurements (n)
astrocytoma	7	56
oligodendroglioma	5	46
ependymoma	4	35
glioblastoma	8	45

class	patients (n)	measurements (n)
astrocytoma	2	18
oligodendroglioma	2	14
ependymoma	1	9
glioblastoma	3	21

Supplementary Table S6. Multiclass necrosis vs. vital tissue data; Above: Training data set. Bottom: Test data set.

class	tumor origin	number of patients	number of measurements
necrosis - carcinoma metastases	NSCLC, colorectal, breast	10	55
vital area - carcinoma metastases	NSCLC, colorectal, breast	17	121
necrosis - glioblastoma	primary brain tumor	14	79
vital area - glioma	primary brain tumors	24	184

class	tumor origin	number of patients	number of measurements
necrosis - carcinoma metastases	NSCLC, colorectal, breast	3	21
vital area - carcinoma metastases	NSCLC, colorectal, breast	6	33
necrosis - glioblastoma	primary brain tumors	5	34
vital area - glioma	primary brain tumors	8	60
